# Phytosynthesis of Zinc Oxide Nanoparticles Using *Ceratonia siliqua* L. and Evidence of Antimicrobial Activity

**DOI:** 10.3390/plants11223079

**Published:** 2022-11-14

**Authors:** Inès Karmous, Fadia Ben Taheur, Nubia Zuverza-Mena, Samira Jebahi, Shital Vaidya, Samir Tlahig, Mohsen Mhadhbi, Mustapha Gorai, Amel Raouafi, Mohamed Debara, Talel Bouhamda, Christian O. Dimkpa

**Affiliations:** 1 Institute of Applied Biology of Medenine, University of Gabes, Medenine 4100, Tunisia; 2 Plant Toxicology and Molecular Biology of Microorganisms, Faculty of Sciences of Bizerte, Jarzouna 7021, Tunisia; 3 Department of Analytical Chemistry, The Connecticut Agricultural Experiment Station, 123 Huntington, New Haven, CT 06511, USA; 4 Laboratory of Analysis, Treatment and Valorization of Environmental Pollutants and Products, Faculty of Pharmacy, University of Monastir, Street Ibn Sina, Monastir 5000, Tunisia; 5 Laboratory of Useful Materials, National Institute of Research and Physicochemical Analysis, Technopole Sidi Thabet, Ariana 2020, Tunisia; 6 Arid Region Institute, Medenine 4100, Tunisia; 7 National Center for Nuclear Science and Technology, Technopole Sidi Thabet, Ariana 2020, Tunisia

**Keywords:** antibacterial activity, antifungal activity, *Ceratonia siliqua* L., green chemistry, phytochemicals, zinc oxide nanoparticles

## Abstract

Carob (*Ceratonia siliqua* L.) is a tree crop cultivated extensively in the eastern Mediterranean regions but that has become naturalized in other regions as well. The present study focused on the green synthesis of zinc oxide nanoparticles (ZnONPs) from Carob and their evaluation for antimicrobial activity in bacteria and fungi. The synthesized ZnONPs showed strong antibacterial activity against *Staphylococcus aureus* ATCC 25 923 (92%). The NPs inhibited the growth of pathogenic yeast strains, including *Candida albicans* ATCC90028, *Candida krusei* ATCC6258, and *Candida neoformans* ATCC14116, by 90%, 91%, and 82%, respectively, compared to the control. Fungal inhibition zones with the ZnONPs were 88.67% and 90%, respectively, larger for *Aspergillus flavus* 15UA005 and *Aspergillus fumigatus* ATCC204305, compared to control fungal growth. This study provides novel information relevant for plant-based development of new and potentially antimicrobial ZnONPs based on extracts. In particular, the development and application of phytogenic nanoparticles enhances the biocompatibility of nano-scale materials, thereby allowing to tune effects to prevent adverse outcomes in non-target biological systems.

## 1. Introduction

Engineered nanomaterials existing in dimensions ranging between 1–100 nanometers (nm) have shown novel applicability in different fields, including food, agriculture, and medicine. Nanoparticles (NPs) possess unique physical properties such as small size, large surface area to volume ratio, electric conductivity, biomimetic property with enhanced catalytic reactivity, thermal conductivity, and non-linear optical performance [1,2]. In particular, the property of increased reactivity has raised the question of whether there are risks of potential toxicity to human and environmental health, and what environmental factors influence such toxicity [3,4]. Therefore, the biosynthesis of NPs has emerged as a preferred approach due to being safer, more cost-effective, biocompatible and eco-friendly, relative to chemically synthesized NPs [5]. The biological pathway for NP synthesis involves use of microorganisms (bacteria, fungi, yeast and algae) and plants. However, compared to microorganisms, plants are free of harmful chemicals and contain natural capping agents, which could enable a greener platform for nanoparticle manufacturing. Moreover, use of plant extracts eliminates the complications involving the use of microbes in nanoparticle synthesis, which involves steps in microorganism isolation, culture maintenance and production in a sterile environment.

Therefore, the use of vegetative organs, cells, and extracts from plant species or marine algae for the synthesis of nanoparticles for deployment in biological applications have generated significant interests, given the relative safety associated in their handling. This contrasts with using microorganisms that may pose pathogenicity concerns, in addition to requiring the maintenance of large cultures in batch reactors [6]. In general, green-synthesis of metallic NPs is based on the capacity of chemical extracts from plants to biologically reduce metallic salts into elemental nanoforms and is influenced by physicochemical parameters such as metal salt concentration, pH and temperature. However, specific outcomes with respect to the synthesized NPs will vary based on the particulars of the chemical composition of the plant extract [7,8,9]. This plant-mediated green chemistry approach consists of three steps: (1) the activation phase, in which metal ions are reduced by the phytoconstituents, followed by the nucleation of reduced metal atoms; (2) the growth phase, wherein small NPs adhere to one another to form larger NPs (Ostwald ripening); and (3) the termination phase, during which NPs attain their shape. Factors playing key roles in this process include (1) the solvent used (in general aqueous extraction); (2) reducing agents (phytoconstituents); (3) the presence of stabilizers including hydroxyl, carboxyl, and amino functional groups in the plant extracts; and (4) capping agents such as surfactants or polymers added to coat NPs to avoid agglomeration or oxidation, and to control the growth of synthesized NPs. Plant secondary metabolites, biomolecules and phytochemicals are potential bioresources for synthesizing nanoparticles. They can serve as reducing, stabilizing, and capping agents in the NP biosynthesis. Such metabolites include flavonoids, terpenoids, alkaloids, phenols, ketones, aldehydes, carboxylic acids, carbohydrates or sugars, proteins, and coenzymes with the potential of reducing metal salts into NPs [10]. These phytocomponents contain various functional groups that are able to reduce metal ions into metal or metal oxide NPs.

Upon biosynthesis, NPs are typically characterized using several techniques. Of these, UV–Visible spectroscopy shows the absorption spectrum of NPs at wavelength range of 200–700 nm. Transmission electron microscopy (TEM) observes the particle size and morphology. Dynamic light Scattering (DLS) determines the size distribution of synthesized spherical metal oxide NPs, as well as the Z-average diameter. Energy-dispersive X-ray spectroscopy (EDX) determines the presence of metal ions in metal oxide synthesized NPs. X-ray diffraction (XRD) allows estimating the average crystalline size of NPs using Scherrer’s equation. Fourier transform infrared spectroscopy (FTIR) determines the nature of the bioactive plant constituents (capping agents) and identifies the functional groups such as alcohols, C–H stretching of methylene group, –C=C–stretching and C=O stretching, –C–H bending vibration of alkanes, –C–O stretching, –C–O–C– vibration, and aromatic C–H bending. Such functional groups are present in phytochemicals such as flavonoids, alkaloids, phenols, proteins and anthracenes. FTIR analysis, therefore, reveals the active involvement of phytochemicals, such as biomolecules, carbohydrates, proteins with functionalized amino groups (–NH_2_) in the reduction of metal ions into NPs [11,12]. 

ZnONPs of varying phytochemical properties and activities have been biosynthesized from different plants parts, including roots, leaves, stems, seeds, buds and fruits [10]. ZnO is an n-type semiconducting metal oxide with a wide range of applications in electronics, optics, and biomedical systems [13]. ZnONPs exhibit semiconducting properties because of its large band gap (3.37 eV) and high exciton binding energy (60 meV) like high catalytic activity, optic, and UV filtering properties. Notably, ZnONPs also find use in a wide range of biomedical applications such as drug delivery, anti-cancer and anti-diabetic therapy, as well as antimicrobial, anti-inflammatory and wound-healing applications [14,15,16]. Also, ZnONPs have piezoelectric and pyroelectric properties [17].

Carob bean (*Ceratonia siliqua* L.) belongs in the Caesalpinioideae sub-family of the legume family, Fabaceae. The carob tree grows to a height of 12–15 m, with a productive life span of more than one hundred years. This legume is an evergreen tree known for its economic and environmental values in the coastal regions of Mediterranean basin and southwest Asia [18]. It has an annual worldwide production of over 315,000 tons of carob products [18]. The Carob pod consists of pulp (90%) and seeds (10%) by weight. In the US, carob is adapted to USDA zones 9–11, which encompass the area between Texas and Florida, and also Hawaii. In much of these places, it is considered a ‘‘neglected’’ crop in the context of contemporary crop science and agronomic research. Carob fruit, flour and syrup have been shown to be rich sources of carbohydrates, proteins and minerals [19]. Carob pulp is rich in antioxidant compounds and nutrients such as fructose, sucrose, glucose and maltose (48–56%), fibers such as cellulose and hemicellulose (18%), and condensed tannins (16–20%) [20]. The plant can be a source of biologically active substances with proven health properties for inclusion in the human diet [18]. Isolated galactomannan from seed gum (30–40% by weight) is indeed used as a valuable stabilizing and thickening additive in the food, pharmaceutical and biotechnology industries [21,22]. In addition, derived products such as laxatives and diuretics are used in medicine [20]. Therefore, carob plant may contain significant amounts of phytochemicals utilizable for the synthesis or development of products with application in agriculture, such as nanoscale agrochemicals. To the best of our knowledge, studies on the use of *C. siliqua* L. pods for the green synthesis of ZnONPs are lacking. Given the rich phytochemical profile of carob and the relevance of Zn in microbial metabolism, the objectives of this study were the biosynthesis of ZnONPs using carob pods, the evaluation of the chemical composition of the carob precursor material, and investigation of the antimicrobial activity of the bio-generated ZnONPs.

## 2. Results 

### 2.1. Phytosynthesis and Characterization of Zinc Oxide Nanoparticles 

**Figure 1:**
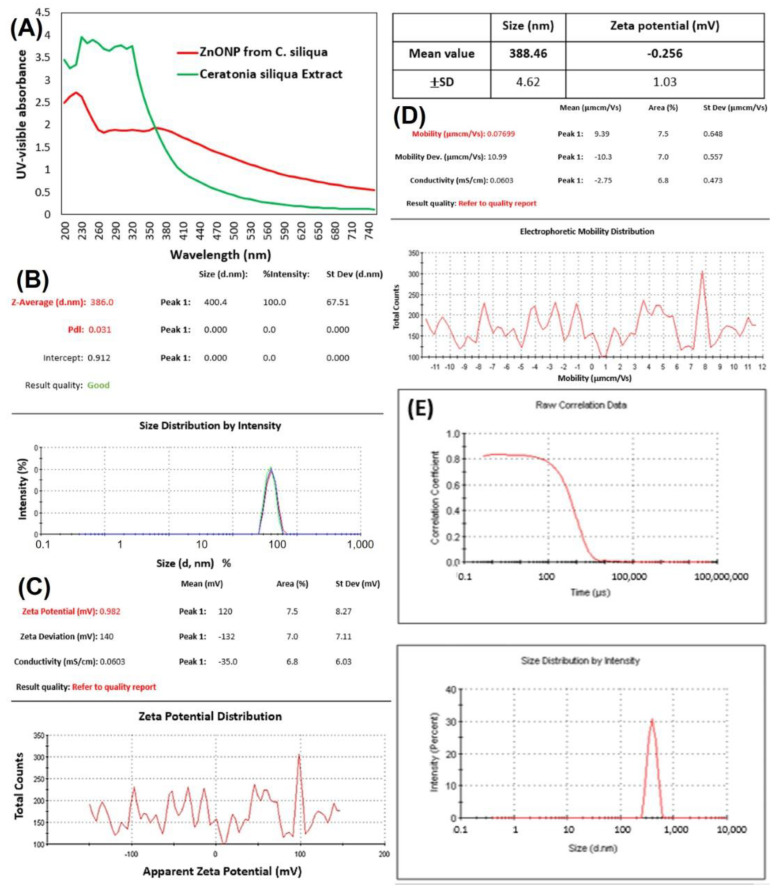
UV-vis absorption spectrum (**A**), size distribution (**B**,**E**), and zeta potential (**C**,**D**) of *C. siliqua* bio-ZnONPs.

**Figure 2:**
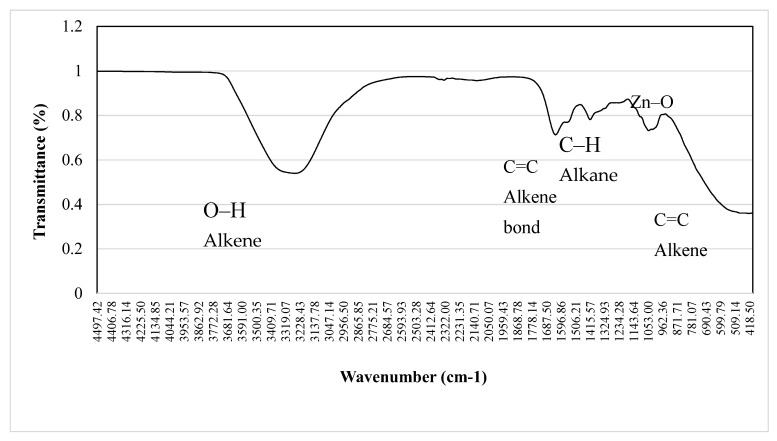
Fourier-transform infrared spectroscopy analysis (FTIR) of *C. siliqua* bio-ZnONPs.

Bio-ZnONPs were synthesized using Zinc acetate as a precursor. The bio-ZnONPs were characterized using several techniques. The biosynthesis of ZnONPs from *C. siliqua* was confirmed by UV–vis absorption (Figure 1). The biosynthesized ZnONPs showed an absorption peak at 360 nm. FTIR spectroscopy (Figure 2) showed the presence of organic functional groups, including carbonyls and hydroxyls attached to the surface of the NPs, as well as other surface chemical residues showing peaks at the range of 400–4500 cm^−1^ at a resolution of 4 cm^−1^. FTIR analysis allowed to ascertain the presence of phytochemicals and functional groups in the plant extract, such as flavones, alcohols, phenols, amines, carboxylic acids, sugars, and ketones, which can interact with the particle surface, and aid in the stabilization of the ZnONPs. The absorption peaks of FTIR spectra at (1) 3550–3200 cm^−1^, (2) 1685–1596 cm^−1^, (3) 1400–1300 cm^−1^, (4) 1415–1380 cm^−1^ and (5) 1000–960 cm^−1^ imply respectively the stretching vibrations of (1) O–H (alkene), (2) C=C stretching, and also (3) N–O stretching, (4) S=O stretching (Figure 1). The peak registered for ZnONP at about 1680 cm^−1^ implies alkene (C=C) stretching, while the peak recorded at 1500 cm^−1^ is assigned to the aromatic bending vibration of alkane group (C–H). 

**Figure 3:**
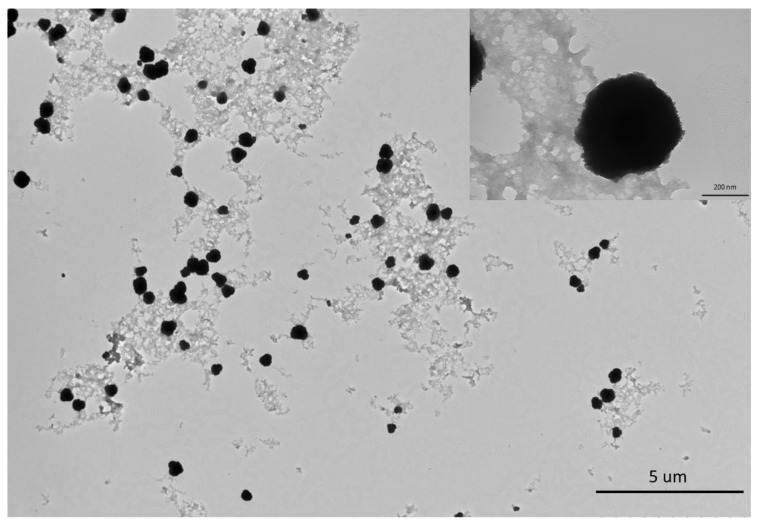
A transmission electron microscopy (TEM) image of *C. siliqua* bio-ZnONPs.

**Figure 4:**
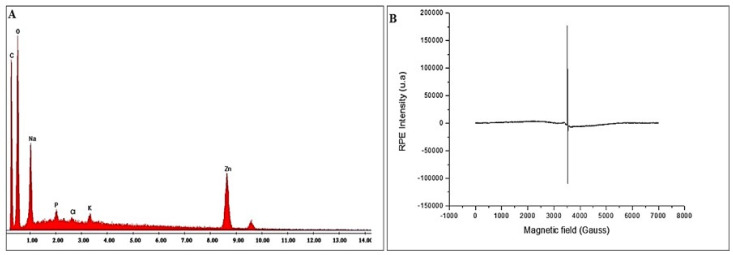
EDAX (**A**) and EPR (**B**) of *C. siliqua* bio-ZnONPs.

In addition, TEM images revealed particles with round morphology and having an average size of 200 µm (Figure 3). EDAX confirmed the main peaks for Zn and O, in addition to peaks corresponding to C and Na, presumably originating from the plant extract (Figure 4A). Electron paramagnetic resonance (EPR) analysis showed that bio-ZnONPs did not demonstrate any magnetic properties (Figure 4B). Further, the particles *contained* amounting to 8.576 ppm ± 1.239, in addition to other minerals, including Na (4.157 ppm ± 0.969), and Ca (2.719 ppm ± 0.304) (Table 1).

### 2.2. Determination of the Chemical Profile of C. siliqua Pods by HPLC-MS

**Figure 5:**
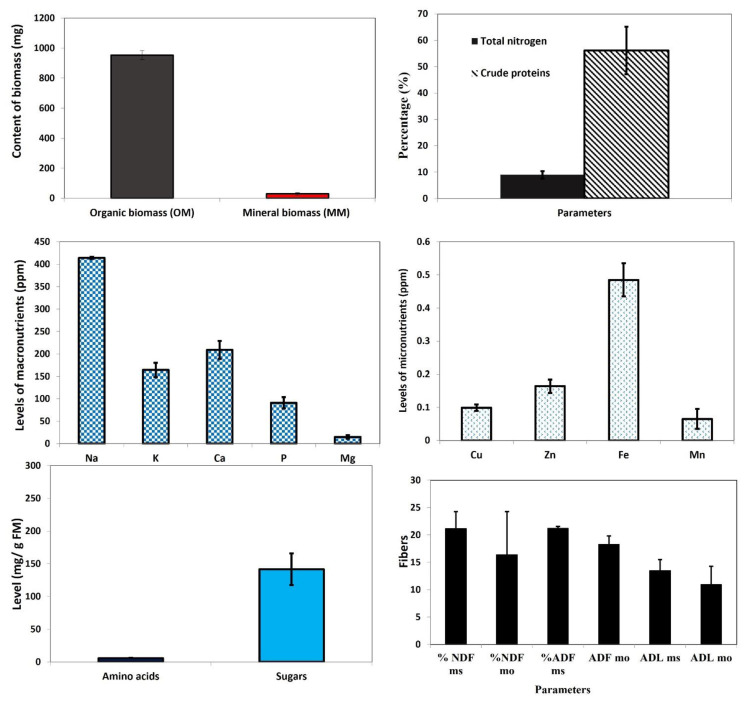
Contents (levels or percentages) in *C. siliqua* of organic biomass (OM), mineral biomass (MM), total nitrogen, crude proteins, macronutrients, micronutrients, amino acids, sugars and fibers. Acid Detergent Fiber (ADF) (cellulose and lignin); Neutral Detergent Fiber (NDF) (cellulose, hemicellulose, and lignin); Acid Detergent Lignin (ADL), Neutral detergent fiber (NDF) and acid detergent fiber (ADF). Data is means of 3 values ±SD. Significance of differences between treatments was estimated at ANOVA (α = 5%) highly significant (*p* value < 0.001).

As this study pioneers the biosynthesis of any metal oxide NPs using *C. siliqua* as the starting plant material, it was important to analyze the chemical composition of the plant material. Figure 5 shows the presence of proteins, sugars, as well as the macronutrients P, Ca, K, Mg, and Na, and the micronutrients Cu, Zn, Fe and Mn. The highest nutrient levels were recorded, in a decreasing order, for Na, Ca, Fe, K, and Zn (Figure 5). 

The carob pods also showed high contents of neutral detergent fiber (NDF; cellulose, hemicellulose, and lignin), followed by acid detergent fiber (ADF; cellulose and lignin), compared to acid detergent lignin (ADL). In addition, flavonoids and phenolic compounds were also detected, revealing high contents of phenolic acids such as quinic acid (49.276 ppm) and gallic acid (16.15 ppm), in addition to polyphenols such as quercetin-3-o-rhamonosic (9.469 ppm), hyperoside (quercetin-3-o-galactoside) (4.019 ppm) and cirsiliol (5.144 ppm). Other compounds detected included luteolin-7-o-glucoside, quercetin, naringin, naringenin, apegenin-7-o-glucoside, apigenin, trans cinnamic, and catechin(+) (Table 2, Figure S1). 

### 2.3. Evaluation of the Antimicrobial Activity of bioZnONP

An assessment of the antimicrobial potential of the *C. siliqua*-based ZnONPs was conducted on bacterial and fungal strains. Table 3 showed the antibacterial activity against *Staphylococcus aureus* ATCC 25 923 (Gram-positive) (zone of inhibition: 12 mm), *versus* no antibacterial activity against *Micrococcus luteus* NCIMB 8166 (Gram-positive), *Salmonella enterica serotype Typhimurium* ATCC 1408 (Gram-negative) and *Escherichia coli* ATCC35218 (Gram-negative). As with bacterial strains, the Bio-ZnONP showed strong inhibitory activity against all tested yeast strains, including *Candida albicans* ATCC90028 and *Candida krusei* ATCC6258 (zone of inhibition: 14 mm) and *Candida neoformans* ATCC14116 (zone of inhibition: 13 mm). Moreover, antifungal effects were recorded against *Aspergillus flavus* 15UA005 with a larger zone of inhibition (17 mm), compared with *Aspergillus fumigatus* ATCC204305 (15 mm), whereas no antifungal activity was observed against *Aspergillus niger* 15UA006 (Table 3).

## 3. Discussion

Plant extracts act as reducing and stabilizing agents in the production of NPs with antimicrobial potential, enabling cost-effective and environment-friendly operations, compared to conventional physical and chemical approaches to synthesizing NPs. In this study we synthesized ZnONPs from carob pods and demonstrated antimicrobial activity with the biosynthesized NPs. The FTIR data indicates that the observed functional groups on the NP surface are the capping ligands derived from *C. siliqua*. The main role of capping ligands is to stabilize the NPs to prevent further growth and agglomeration which modulates their bioactivity [23]. The capping process by the functional groups can also occur during the green synthesis process, thereby controlling growth in situ [24]. In this case, the phytochemicals such as favones, alcohols, phenols, amines, carboxylic acids, sugars and ketones can interact with the zinc surface and aid in the stabilization of NPs. Water soluble phytoconstituents such as flavones, quinones, and organic acids that are available in the plant extract could have contributed towards an instantaneous reduction of zinc ions in the synthesis process. Overall, the stretching vibrations in FTIR spectra of bioZnONPs, confirm that C. *siliqua* phytocompounds serve as coating, capping, and stabilizing agents for ZnONPs; and that the Zn–O frequencies observed for the bioZnONP are in accordance with values in the literature [25,26]. Besides, the relative chemical composition of the plant material suggests that sugars from plants may be responsible for the reduction of metal salts into the NPs, a likelihood corroborated by previous findings with other metallic NPs [27,28]. However, amino acids and proteins may also be involved, but likely to a lesser degree. In addition, bio-capping may be achieved by the carboxylic and phenolic acids present in the carob pod extract. These capping agents are important for preventing agglomeration and controlling the biosynthesis. Also, Zinc reduction achieved through the phytoconstituents (flavonoids or other polyphenols) present in *C. siliqua* may be considered as an important progress in this path. In this regard, the screening of the polyphenols and phenolic acids present in *C. siliqua* suggests some of the main phenolic compounds that may have been involved in the capping and stabilization of NPs, notably flavonoids and phenolic compounds. 

Findings on the antimicrobial activity of the biosynthesized ZnONPs agree with other reports on the efficacy of green synthesized metal oxide NPs, including ZnONPs, as antibacterial agents [29,30,31,32,33]. Gupta et al. [34] also demonstrated significant antibacterial activity of ZnONPs against similar strains, including *Staphylococcus aureus* MTCC 9760, *Streptococcus pyogenes* MTCC 1926, *Bacillus cereus* MTCC 430, *Pseudomonas aeruginosa* MTCC 424, *Proteus mirabilis* MTCC 3310 and *E. coli* MTCC 40. These results are also in line with those of Renganathan et al. [30] who showed that biologically synthesized silver NPs (AgNPs) exhibited antibacterial activity against *Staphylococcus aureus* and *E. coli*, with larger inhibition zone formation against *S. aureus* (9.25 mm), compared with that of *E. coli* (6.75 mm), in addition to antifungal activity against *Aspergillus flavus* and *Candida albican* (with superior inhibition against *A. flavus* (zone of inhibition: 7 mm) compared with *C. albicans* (zone of inhibition: 5.75 mm). Similar antimicrobial and anticancer activities were reported for ZnONPs and AgNPs [12,35,36]. Notably, *Aspergillus flavus* is a plant pathogen; research to understand the effect of the carob-synthesized ZnONPs is ongoing in an *Aspergillus*-plant pathosystem, which would demonstrate the applicability of work reported in this manuscript in plant production.

Drug or agrochemical resistance by strains of human and plant pathogenic microbes have increased in recent times. Hence, alternative strategies to control microbial infections in biological systems are required. In this wise, studies to establish nanoscale materials such as ZnONPs as viable options for novel antimicrobials have gained prominence in recent times. Taken together, therefore, data from the current study contribute to the search for enhanced efficiency biogenic antibacterial and antifungal agents. Increasingly, NPs continue to be assessed as antibiotics because of their strong antimicrobial activities [37], while plant extract have already been used as reducing antibacterial and antifungal agents [22]. The combination of NPs and plant derived extracts therefore provides a synergy among specific plant constituents and nanosized particles for biological activity. The biosynthesized plant extract-derived metal/metal oxide NPs may act via their ability to inhibit key enzymes, such as bacterial DNA gyrase and fungal cytochrome P450 (*C. albicans*) and dihydrofolate reductase (*A. flavus*), thus resulting in inactivation of bacterial or fungal microorganisms [34].

The fundamental shortcoming of research in the field of phytosynthesis of NPs is that findings are sometimes conflicting, and there are no consistent outcomes. This is because to a considerable extent, the findings differ substantially because phytochemical composition and quantities vary from plant to plant. Additionally, the size and morphology of NPs are controlled by certain phytochemicals and chemistry of the specific precursor salt. To clarify the mechanism, it is necessary to identify the exact phytochemicals participating in the NP biosynthesis process. However, in attempting to understand the correlations between the plant extract composition, the phytosynthesis, and NP morphology and surface chemistry, there is a lack of appropriate plant extract characterization in accordance with the common phytoconstituents. In this regard, there is need, as demonstrated in the current work, to identify plants with superior biochemical arsenals that can be appropriately characterized, quantified and deployed in NP synthesis to provide novel agrochemicals that can be applied in improving and sustaining crop production in the face of increased events of disease outbreaks that devastate crop yield. 

## 4. Materials and Methods 

### 4.1. Biosynthesis and Characterization of Biosynthesized C. siliqua *L*. Bio-ZnONPs

Carob (*C. siliqua* L.) pods were dried at room temperature for one month. Subsequently, pods were crushed mechanically using a kibbler, and separated from seeds. Pods were then ground into powder in a mixer. 5–10 g of the pod powder was added to distilled water for 1 h under heating at 60 °C, followed by filtration using Whatman filter paper (diameter 0.9 cm) and the filtrate was stored at 4 °C. A volume of 100 mL of plant broth was mixed with 0.1 M zinc acetate dehydrate (*v*/*v*). The mixture was incubated in a boiling water bath at 80 °C for 2 h. The formed bio-ZnONPs precipitates were collected by centrifugation at 8000× *g* for 10 min. Supernatants were eliminated, and pellets were collected and washed 3 times with distilled water to remove impurities. This product I hereinafter referred as “*C. siliqua.* bio-ZnONPs”. Characterization of synthesized materials was done using standardized analytical techniques. The formation of bio-ZnONPs was confirmed by measuring UV-visible absorption spectrum using a Spectra Max M2 spectrophotometer. Fourier Transform Infra-Red (FTIR) spectra was recorded by using a Bruker Equinox 55 spectrometer. The particle size distribution and the surface area of the samples were determined at the wavelength of 400–4000 cm^−1^, and a resolution of 2 cm^−1^ using a laser analyzer Malvern Mastersizer 2000. Dynamic light-scattering (DLS) was used to determine the particle size distribution, and the zeta potential, which represents the charge on the NPs’ surface. Transmission electron microscopy (TEM) images was used to characterize particle morphology and size (Hitachi HT 7800, 120 kV). EDX analysis was performed using a FEI Quanta 200 environmental scanning electron microscopy (SEM; Leuven Belgium, PHILIPS/FEI), operating at 20 kV. For magnetic properties of NPs, EPR spectra were obtained at room temperature on a Bruker ER-200D spectrometer operating at 9.8 GHz X-Band frequencies with modulation amplitude of 0.2 mT, modulation frequency of 100 khz, sweep width of 210 mT and microwave power of 63 mW.

### 4.2. Elemental Analysis of Bio-ZnONPs

The content of Zn and other mineral elements in *C. siliqua* pods was measured using inductively coupled plasma optical emission spectroscopy (iCAP 6000; DAC0083, Thermo Fisher Scientific). ICP-OES method was validated using blank, 0.1 ppm, 1 ppm, and 10 ppm standard solutions (quality control standard 7 and 21, SPEX CertiPrep, Metuchen, NJ, USA; prepared in 2% nitric acid). Yttrium (10 ppm) was used as an internal standard. A 30 s rinse with DI water was carried out between samples, and continuing calibration verification sample was done each 15 samples. Results were expressed in ppm. 

### 4.3. Determination of Dry Biomass (DM), Organic Biomass (OM), Mineral Biomass (MM) and Total Nitrogen

Fresh pods were placed at 40 °C for 24 h to obtain dry biomass (DM). Mineral biomass (MM), referred as ash, was determined after combustion at 550 °C in oven for 6 h and expressed as a percentage of the DM. Organic biomass (OM) was calculated using the formula DM-MM.

The total nitrogen content in dry pods (0.5 g of DM) was determined using Kjeldahl [38], and used as a surrogate for protein content. A control reaction (in the absence of sample) was included as a reference. The percentage of nitrogen was calculated as %N = 100 × (VHCl for sample-V0) × 0.1 (N) × 0.140/m (g), where V0 refers to the volume of HCl used in titration. The concentration of crude proteins was then calculated as CP = %N × 6.25. 

### 4.4. Elemental Analysis 

Determination of mineral elements by atomic absorption spectrometry (AAS). Pods of *C. siliqua* were dried at 120 °C in oven and ground in a mortar. One gram of dry biomass was subjected to mineralization at 500 °C in oven for 6 h. The obtained ashes were solubilized in HCl (4:1, concentrated HCl: distilled H_2_O) under heating at approximately 200 °C. Homogenates were then filtered twice with ash-free filter papers and the final volumes of filtrates were adjusted to 50 mL. Atomic absorption spectrometry (AAS; Thermo SCIENTIFIC, Type iCE3500AA System, NC942350023500, Ser No C103500104) was used to determine the contents of essential mineral elements in the pods. Total phosphorus was estimated using the vanado-molybdic reaction solution [39]. The solution consisted of 50 mL of ammonium heptamolybdate (NH_4_)_6_Mo_7_O_24_.4H_2_O (5 g dissolved in H_2_O and 0.5 mL ammoniac (d: 0.91)), 50 mL ammonium monovanadate NH_4_VO_3_ (0.1175 g) and 3.35 mL nitric acid (HNO_3_) in 200 mL total volume. A mass of 0.1175 g of ammonium monovanadate (NH_4_VO_3_) was dissolved in 20 mL of hot H_2_O. Potassium dihydrogen phosphate (KH_2_PO_4_) was used to prepare standard concentrations up to 40 μg/mL. Pods were dried at 120 °C, then mixed with 1 mL of vanado-molybdic reactional solution. After homogenization, the mixture was allowed to stand for 10 min at 20 °C, after which absorbance was read at 730 nm using a UV-VIS spectrophotometer (Model T80, 25-1884-01-0002, 180W).

### 4.5. Measurement of Pod Fiber Content

The protocol of ANKOM 220 Fiber bag method [ANKOM Technology [Filter Bag Technique in Feeds (for A200 and A200I)] was used for the determination of fiber contents in cell walls (cellulose, hemicellulose and lignin); Acid Detergent Fiber (ADF) (cellulose and lignin); Neutral Detergent Fiber (NDF) (cellulose, hemicellulose, and lignin); and Acid Detergent Lignin (ADL). The levels of cellulose, hemicellulose, lignin, crude cellulose (CB), NDF, and ADF were estimated using the method of Van Soest [40]. To measure ADF, an acid detergent solution (ADS) was used, in which 20 g of cetyltrimethyl ammonuim bromure (CTAB) was dissolved in H_2_SO_4_ (1 N). Subsequently, one gram of dry *C. siliqua* pods (Pe) was boiled with ADS for 60 min, followed by filtration and rinsing with acetone, and drying at 105 °C for 24 h (1st desiccation) (P1). A volume of 30 mL of ADSL (acid detergent lignin solution) prepared by placing sample bags in a flask containing 350 mL of sulfuric acid H_2_SO_4_ 72% by weight. After 3 h, the mixture was filtered and rinsed with hot distilled water, then dried at 105 °C for 24 h (2nd desiccation) (P2). Samples were then oven-dried at 500 °C for 3 h (incineration) P3. Calculations were made using the following formulas (manufacturer instructions):

% **ADF** (as_received basis) = [**100** × (**W_3_** − (**W_1_** × **C_1_**))]/**W_2_**.

With; **W1**: bag tare weight; **W_2_**: sample weight; **W3**: dried weight of bag with fiber after extraction process; and **C1**: blank bag correction (running average of final oven dried weight divided by original blank bag weight.

**ADL** (as_received basis) = [**100** × (**W_3_** − (**W_1_** × **C_1_**))]/**W_2_**.

**ADL_DM_** (DM basis) = [**100** × (**W_3_** − (**W_1_** × **C_1_**))]/**W_2_** × **DM**.

**ADL_OM_** (DM basis) = [**100** × (**W_4_** − (**W_1_** × **C_2_**))]/**W_2_** × **DM**.

With: **W_1_**: bag tare weight; **W_2_**: sample weight; **W_3_**: weight after extraction process; W_4_: weight of organic matter (OM) (weight loss on ignition of bag and fiber residue); **C_1_**: blank bag correction (final oven_dried weight/original blank bag weight); **C_2_**: ash corrected blank bag (loss of weight on ignition of bag/original blank bag); MD: dry matter. 

To measure NDF, sodium dodecyl sulfate (SDS; C12H25NaSO4), a neutral detergent solution (NDS) was used, in which one gram of biomass (Pe) was added to 100 mL de solution NDS, and boiled for 60 min, after which the homogenate was filtered and rinsed with distilled H_2_O, and bags were placed in a flask containing pure acetone. The mixture was then dried at 105 °C for 24 h (desiccation) (P1), then in an oven at 500 °C for 3 h (incineration) (P2).

% NDF (as_received basis) = [**100** × (**W_3_** − **(W_1_** × **C_1_**))]/**W_2_**.

With; **W1**: bag tare weight; **W_2_**: sample weight; **W3**: dried weight of bag with fiber after extraction process; and **C1**: blank bag correction (running average of final oven dried weight divided by original blank bag weight. 

### 4.6. Assay of Free Amino Acids and Total Soluble Sugars

Fresh carob pods were extracted and stored at −20 °C. Subsequently, homogenization (1:10, *w*/*v*) was performed in 80% ethanol at 70 °C (1:10, *w*/*v*), followed by centrifugation at 8000× *g* for 20 min at 4 °C. The obtained supernatant speculated to contain free amino acids and total soluble sugars were analyzed to quantify these compounds. For free amino acids, an aliquot of extract was added to 0.9 M citric acid (pH 5.2) and ninhydrin solution 1.5% (prepared in ethanol 60% /H_2_O, *v*/*v*, in the presence of ascorbic acid). The reaction mixture was incubated at 100 °C for 15 min and then allowed to cool at 4 °C for 5 min. After the immediate addition of ethanol 60%, absorbance of the mixture was read at 570 nm using glycine as standard [41]. For total soluble sugars, an aliquot of the extract was mixed with a 0.2% anthrone solution in concentrated sulfuric acid. After incubation at 100 °C for 15 min and cooling at 4 °C for 5 min, absorbance was measured at 625 nm using glucose as standard [42].

### 4.7. Determination of the Phenolic Compounds by HPLC-MS

Dried carob pods were ground into powder, after which a powder mass of 5 g was homogenized in 80% methanol (1:10, *w*/*v*), centrifuged at 8000× *g* for 20 min, and the supernatants filtered through a 0.45 µm cellulose acetate filter (Millipore). Phenolic compounds were analyzed by Liquid Chromatography-Mass Spectrometry using a Shimadzu UFLC XR system (Kyoto, Japan), consisting of a CTO-20 AC column, a LC-20ADXR binary pump and a quadrupled 2020 detector system. The chromatography was performed using an Inertial ODS-4 C18 3 µm column L150 × 3.0 mm i.d, at column temperature set at 40 °C. The injection volume was 20 µL, and the flow rate was 0.5 mL min^−1^. The mobile phases were: (A) 5% methanol, containing 0.15% acetic acid and (B) 50% ACN, containing 0.15% acetic acid. Elution proceeded in a linear gradient system of 0.01–14 min from 10% to 20% (B); 14–27 min from 20% to 55% (B); 27–37 min from 55% to 100% (B); 37–45 min with 100% (B), followed by column washing and equilibration for 45–50 min with 10% (B). Dissolving line temperature was 275 °C, mobilizing gas flow was 1.50 mL/min, the drying gas was set at 15.00 mL/min, and Temperature of Heat block was set at 450 °C. LC-ESI (-) MS mass spectra [M-H]-were acquired using Lab Solutions software. Phenolic acids were identified by comparison with retention time of the standards of polyphenolic compounds. The lab standards used were LGC and Sigma Aldrich.

### 4.8. Evaluation of the Antibacterial Activity of bioZnONP

The bacteria for the evaluation consisted of Gram positive (*Staphylococcus aureus* ATCC 25 923 and *Micrococcus luteus* NCIMB 8166) and Gram negative (*Salmonella enterica serotype Typhimurium* ATCC 1408 and *Escherichia coli* ATCC35218) strains. Bacterial culture was carried out on nutritive gelose at 37 °C for 24 h. Subsequently, selected colonies were inoculated into suspensions of 10 mL volume of sterile physiologic water and then mixed for 5 min. Growth was monitored by measuring the optic density (OD) of the culture at 600 nm and adjusted up to OD value 0.5. A volume of 1 mL of the bacterial culture was used on Muller Hinton gelose in a petri dish. Then, 100 μL (10 mg/mL) of carob Bio-ZnONPs was added into 6 mm diameter of wells. Petri dishes were incubated at 37 °C for 24 h. The diameter of inhibition zone was measured using digital caliper. These experiments were carried out in triplicates under sterile conditions. 

### 4.9. Evaluation of Activity against Yeast Growth

Yeast strains consisting of *Candida albicans* ATCC90028, *Candida krusei* ATCC6258 and *Candida neoformans* ATCC14116 were used for this evaluation. Culture was performed on Sabouraud gelose at 37 °C for 48 h. Selected colonies were inoculated into suspensions of 10 mL volume of sterile physiologic water and mixed for 5 min. Growth was monitored by measuring the optic density (OD) of the culture at 600 nm and adjusted up to OD value 0.5. One mL of the yeast suspension was inoculated on Sabouraud gelose and grown for 30 min at 37 °C. Then, 6 mm of diameter wells were prepared on gelose. A volume of 1 mL of the bacterial culture was used on Muller Hinton gelose in a petri dish. Then, 100 μL (10 mg/mL) of carob Bio-ZnONPs was added into 6 mm diameter of wells. Following incubation for growth at 37 °C for 48 h, diameters of inhibition zone surrounding the sample zone were measured and expressed in mm. Three replicates were involved with experiments conducted under sterile conditions. 

### 4.10. Evaluation of Antifungal Activity 

The fungal strains, *Aspergillus flavus* 15UA005, *Aspergillus niger* 15UA006 and *Aspergillus fumigatus* ATCC204305 were cultured on Sabouraud gelose in Falcon tubes of 15 mL at 25 °C for 7 days. Spores of the fungal strain were transferred into suspensions of peptone water and counted up to 106 spores/mL. One mL of fungal suspension was inoculated on Sabouraud gelose in petri dish and incubated for growth at 25 °C for 30 min. Then, 6 mm of diameter wells were prepared on gelose. A volume of 100 μL (10 mg/mL) of the Bio-ZnONPs sample was added into the wells. Following incubation for yeast growth at 25 °C for 48 h, the diameters of inhibition zone surrounding the sample zone was determined and expressed in mm. The test was carried out in triplicates under sterile conditions. 

### 4.11. Statistical Analysis

Statistical analyses were carried out using SPSS 20.0 and Xlstat version 9.0 software 2014 SPSS(Statistical Product and Service Solutions) developed by SPSS Inc. under IBM company, Chicago, Illinois. Data were subjected to analysis of variance (two ways) ANOVA at α = 0.05%. Means were compared using Duncan multi-range test at 5%.

## 5. Conclusions

We demonstrate for the first time that extracts from the carob plant, *C. siliqua* L., can be used for the synthesis if ZnONPs; and that the ZnONPs can effectively be used as an alternative antimicrobial agent. These results portend a demonstration of a novel tool for developing more efficient solutions against antibacterial and antimicrobial resistance in both human and plant pathogens. However, further research is required to assess the mechanisms of action and toxicity of the phytosynthesized NPs, including for untargeted microbes, especially in plant-pathosystems. Also, studies to establish optimal dose are required, compared to conventional antimicrobials. Furthermore, a rapid, cost-effective, environmentally-friendly method for ZnONPs synthesis was demonstrated, which can be used as a rapid screening strategy agent against different microbial species with relevance in human and agricultural health. 

## Figures and Tables

**Table 1:** Determination of levels of Zn and other mineral elements in *C. siliqua.* bio-ZnONPs.

Minerals	Ca	Cu	Fe	K	Mg	Mn	Mo	Na	P	Pb	S	Zn
Mean value	2.719	0.006	0.052	0.209	0.165	0.00243	0.002	4.157	0.1	0.032	0.927	8.576
±SD	0.304	0.004	0.011	0.035	0.023	0.0006	0.003	0.969	0.018	0.049	0.214	1.239

**Table 2:** Identification and quantification of the phenolic compounds by HPLC-MS.

Phenolic Compounds	Level (ppm)
Quinic acid	49.276
Gallic acid	16.15
Protocatechuic acid	0.032
p-coumaric acid	0.195
transferulic acid	0.037
Luteolin-7-o-glucoside	0.278
Quercetin	0.135
Quercetin (quercetin-3-o-rhamonosic)	9.469
Hyperoside(quercetin-3-o-galactoside)	4.019
Naringin	2.155
Naringenin	1.276
Apegenin-7-o-glucoside	0.401
Apegenin	0.449
trans cinnamic	1.816
cirsiliol	5.144
catechin(+)	0.873
Epicatechin	0.041
syringic acid	0.141

**Table 3:** Antimicrobial activities of *C. siliqua*. bio-ZnONPs.

**Bacterial Strains**	**Antibacterial Activity** **Inhibition Diameter (mm)**
*Staphylococcus aureus* ATCC 25 923	12 ± 0.71
*Micrococcus luteus* NCIMB 8166	0
*Salmonella enterica sérotype Typhimurium* ATCC 1408	0
*Escherichia coli* ATCC35218	0
**Yeast strains**	**Activity against yeast growth** **Inhibition diameter (mm)**
*Candida albicans* ATCC90028	14 ± 0.71
*Candida krusei* ATCC6258	14 ± 0.00
*Candida neoformans* ATCC14116	13 ± 0.71
**Fungal strains**	**Antifungal activity** **Inhibition diameter (mm)**
*Aspergillus flavus* 15UA005	17 ± 0.71
*Aspergillus niger* 15UA006	0
*Aspergillus fumigatus* ATCC204305	15 ± 0.71

## Data Availability

The datasets generated during and/or analyzed during the current study are available from the corresponding author upon reasonable request.

## Supplementary Materials

The following supporting information can be downloaded at: https://www.mdpi.com/article/10.3390/plants11223079/s1, Figure S1: Chromatographs of total phenolic compounds analyzed by HPLC-MS.
