# Supplementary material for: Phytosynthesis of Zinc Oxide Nanoparticles Using Ceratonia siliqua L. and Evidence of Antimicrobial Activity
Source: Plants (Basel). 2022 Nov 14;11(22):3079. doi: 10.3390/plants11223079 (PMC9695060; doi:10.3390/plants11223079)
Supplement: Supplementary file 1 [file plants-11-03079-s001.zip › plants-1949311-supplementary.pdf]

Phytosynthesis of zinc oxide nanoparticles using *Ceratonia siliqua* L. and evidence of antimicrobial activity

Inès Karmous[1,2,3], Fadia Ben Taheur[4], Nubia Zuverza-Mena[3], Samira Jebahi[1,5], Shital Vaidya[3], Samir Tlahig[1,6], Mohsen Mhadhbi [5], Mustapha Gorai [1], Amel Raouafi[7], Mohamed Debara [6], Talel Bouhamda [6] and Christian O. Dimkpa [3] *

1. Institute of Applied Biology of Medenine (ISBAM), University of Gabes, 4100, Medenine, Tunisia;
2. Plant Toxicology and Molecular Biology of Microorganisms, Faculty of Sciences of Bizerta, Zarzouna, Tunisia.
3. The Connecticut Agricultural Experiment Station. 123 Huntington, New Haven, CT 06511, Connecticut, USA.
4. Laboratory of Analysis, Treatment and Valorization of Environmental Pollutants and Products, Faculty of Pharmacy, University of Monastir, Rue Ibn Sina, Monastir 5000, Tunisia.
5. Centre National des Sciences et Technologies Nucléaires (CNSTN). Rte de Tunis 2020, Sidi Thabet Ariana, Tunisia.
6. Arid Region Institute, 4100, Medenine, Tunisia.
7. Institut National de Recherche et d'Analyse Physico-chimique (INRAP), Tunisia.

* Correspondence: Corresponding author: C. Dimkpa: Email: Christian.Dimkpa@ct.gov

MS Quantitation
191,00(-)@1

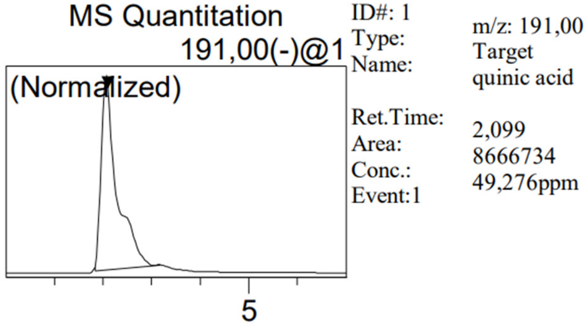

ID#: 1
Type: Target
Name: quinic acid
m/z: 191,00

Ret.Time: 2,099
Area: 8666734
Conc.: 49,276ppm
Event:1

MS Quantitation
169,00(-)@1

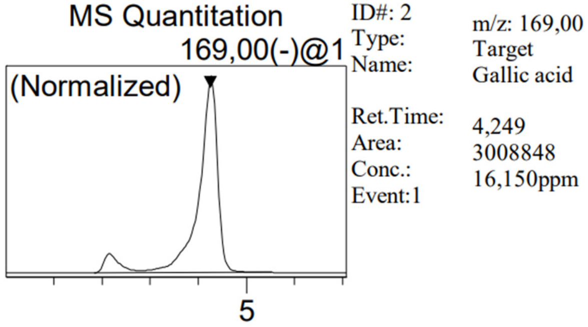

ID#: 2
Type: Target
Name: Gallic acid
m/z: 169,00

Ret.Time: 4,249
Area: 3008848
Conc.: 16,150ppm
Event:1

MS Quantitation
197,00(-)@1

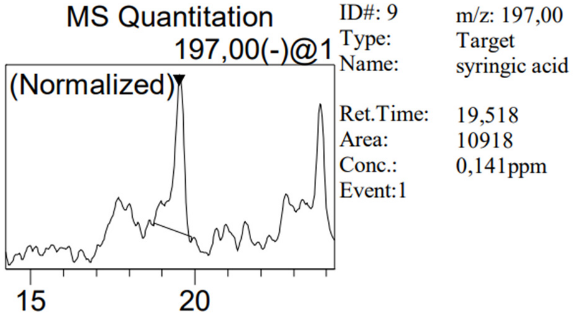

ID#: 9
Type: Target
Name: syringic acid
m/z: 197,00

Ret.Time: 19,518
Area: 10918
Conc.: 0,141ppm
Event:1

MS Quantitation
153,00(-)@1

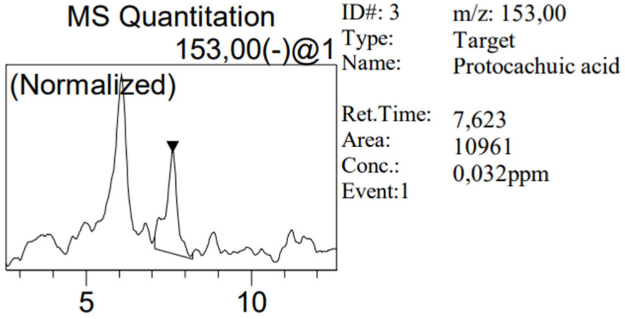

ID#: 3
Type: Target
Name: Protocachuic acid
m/z: 153,00

Ret.Time: 7,623
Area: 10961
Conc.: 0,032ppm
Event:1

MS Quantitation
289,00(-)@1

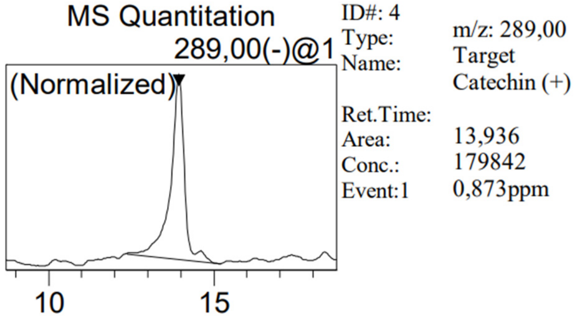

ID#: 4
Type: Target
Name: Catechin (+)
m/z: 289,00

Ret.Time: 13,936
Area: 179842
Conc.: 0,873ppm
Event:1

MS Quantitation
289,00(-)@1

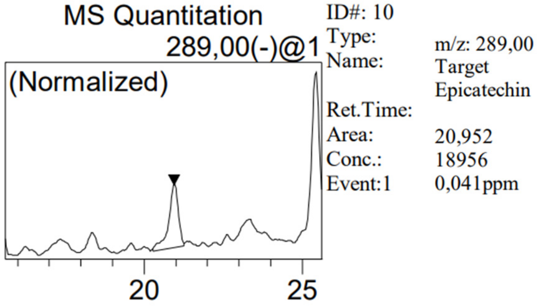

ID#: 10
Type: Target
Name: Epicatechin
m/z: 289,00

Ret.Time: 20,952
Area: 18956
Conc.: 0,041ppm
Event:1

MS Quantitation
163,00(-)@1

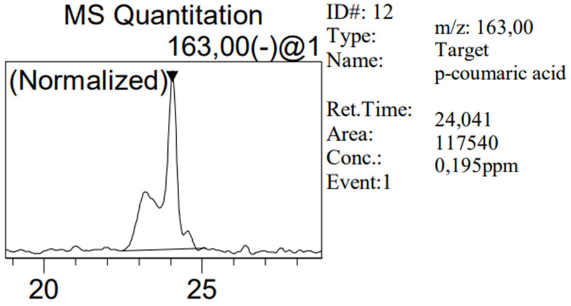

ID#: 12
Type: Target
Name: p-coumaric acid
m/z: 163,00

Ret.Time: 24,041
Area: 117540
Conc.: 0,195ppm
Event:1

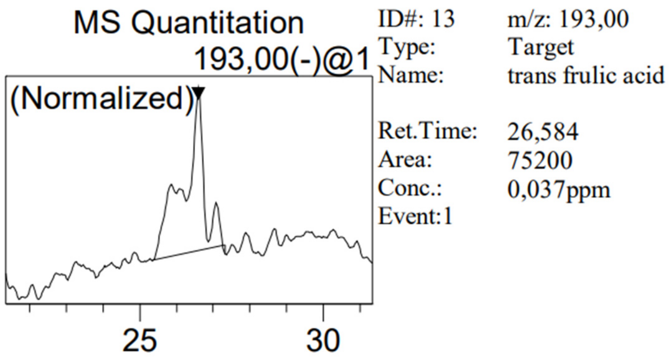

ID#: 13
Type:
Name:

Ret.Time:
Area:
Conc.:
Event:1

m/z: 193,00
Target
trans frulic acid

26,584
75200
0,037ppm

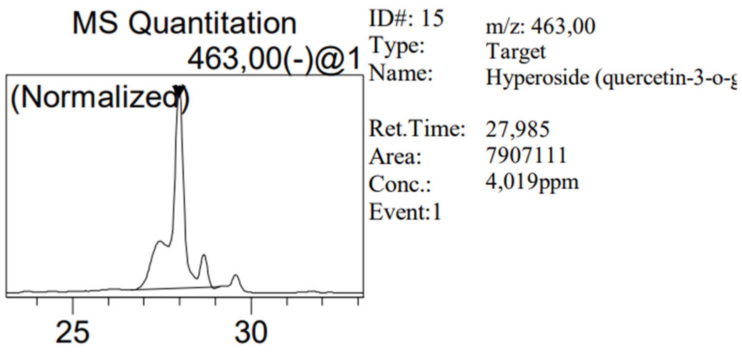

ID#: 15
Type:
Name:

Ret.Time:
Area:
Conc.:
Event:1

m/z: 463,00
Target
Hyperoside (quercetin-3-o-g

27,985
7907111
4,019ppm

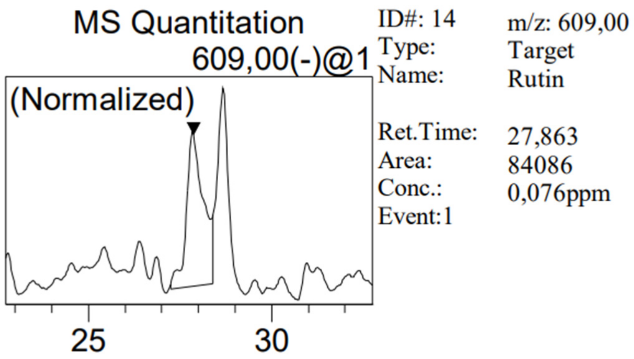

ID#: 14
Type:
Name:

Ret.Time:
Area:
Conc.:
Event:1

m/z: 609,00
Target
Rutin

27,863
84086
0,076ppm

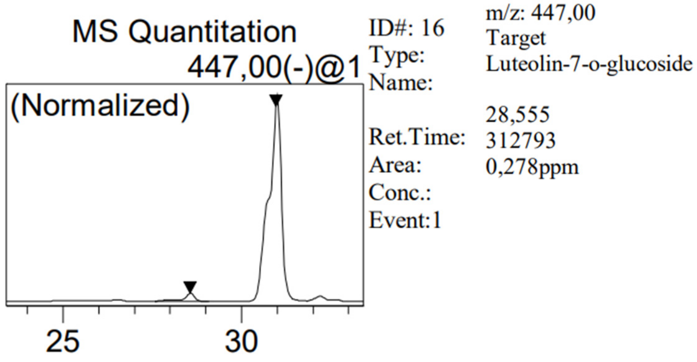

ID#: 16
Type:
Name:

Ret.Time:
Area:
Conc.:
Event:1

m/z: 447,00
Target
Luteolin-7-o-glucoside

28,555
312793
0,278ppm

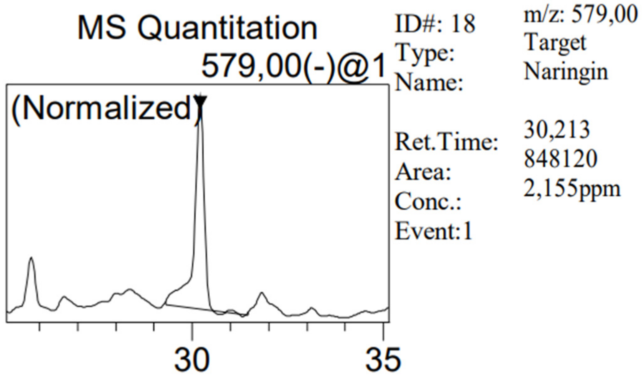

ID#: 18
Type:
Name:

Ret.Time:
Area:
Conc.:
Event:1

m/z: 579,00
Target
Naringin

30,213
848120
2,155ppm

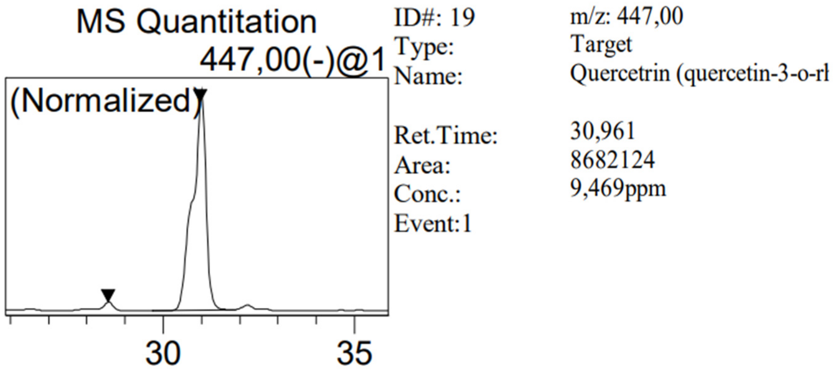

ID#: 19
Type:
Name:

Ret.Time:
Area:
Conc.:
Event:1

m/z: 447,00
Target
Quercetrin (quercetin-3-o-rl

30,961
8682124
9,469ppm

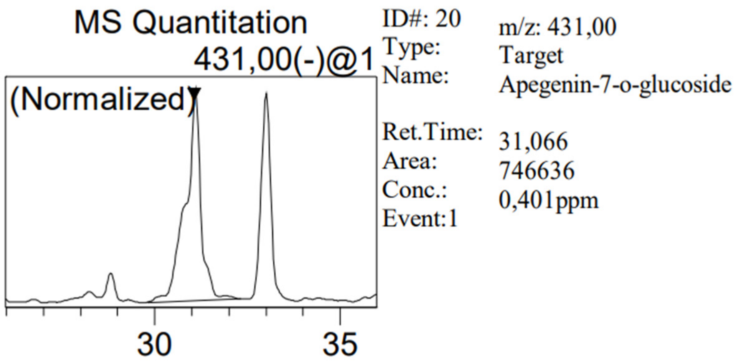
MS Quantitation
431,00(-)@1
(Normalized)
30
35
ID#: 20
Type:
Name:
m/z: 431,00
Target
Apegenin-7-o-glucoside
Ret.Time:
Area:
Conc.:
Event:1
31,066
746636
0,401ppm

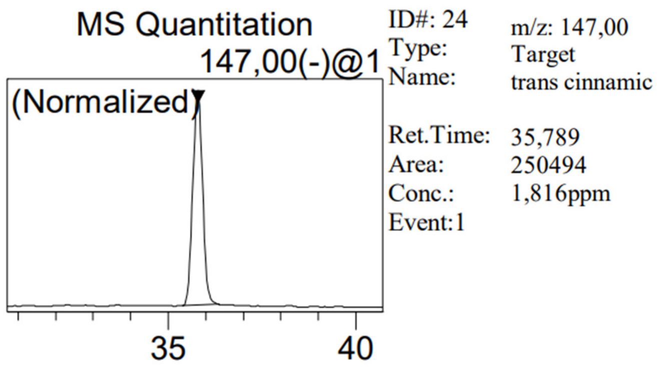
MS Quantitation
147,00(-)@1
(Normalized)
35
40
ID#: 24
Type:
Name:
m/z: 147,00
Target
trans cinnamic
Ret.Time:
Area:
Conc.:
Event:1
35,789
250494
1,816ppm

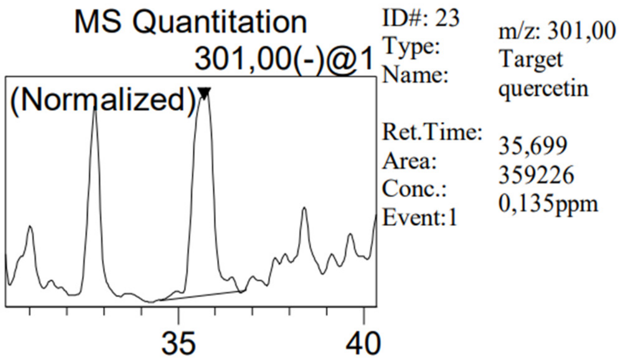
MS Quantitation
301,00(-)@1
(Normalized)
35
40
ID#: 23
Type:
Name:
m/z: 301,00
Target
quercetin
Ret.Time:
Area:
Conc.:
Event:1
35,699
359226
0,135ppm

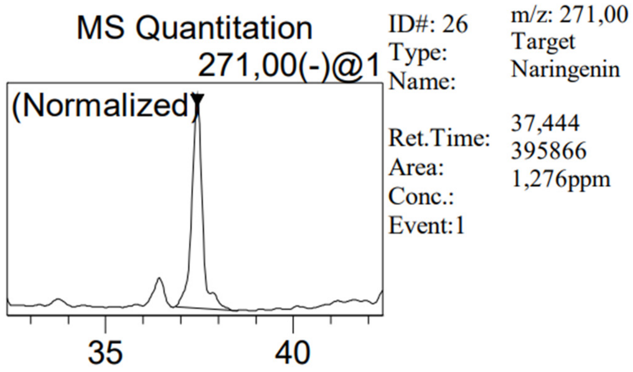
MS Quantitation
271,00(-)@1
(Normalized)
35
40
ID#: 26
Type:
Name:
m/z: 271,00
Target
Naringenin
Ret.Time:
Area:
Conc.:
Event:1
37,444
395866
1,276ppm

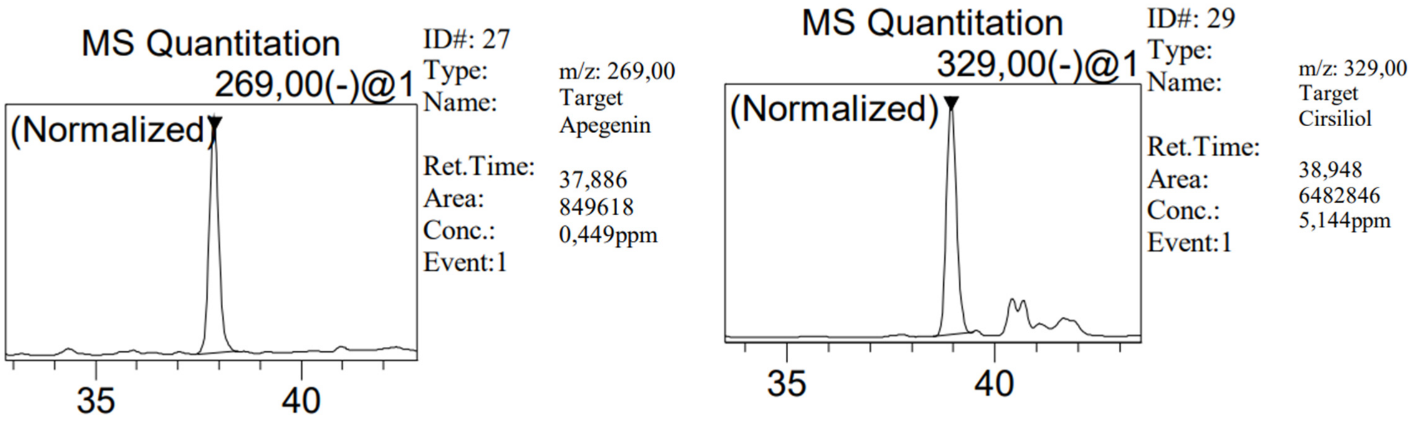

Figure S1 Chromatographs of total phenolic compounds analyzed by HPLC-MS.
